# A Practical Treatise on Sexual Disorders in the Male and Female

**Published:** 1906-03

**Authors:** 


**A Practical Treatise on Sexual Disorders in the Male and Female.** By **Robert W. Taylor,** A.M., M.D., Clinical Professor of Genitourinary and Venereal Dseases in the College of Physicians and Surgeons (Columbia University), New York. Third edition, enlarged and revised. Octavo, 575 pages with 130 engravings and 16 colored plates. Philadelphia and New York. Lea Brothers & Co. 1905. (Cloth, $3.00 net).

The value of a work on a special subject is shown by an honest demand for new editions, which is quite true of this excellent book on the sexual disorders of the male and female. The author apparently was not satisfied with the conditions of the second edition, for in this latest volume he practically presents a new book, the whole text having been gone over, revised, amplified and brought up to the very last moment of research. Dr. Taylor's efforts to produce as nearly a perfect book as may be, have been most ably seconded by his publishers, who have given the work a most artistic appearance. There is a more satisfying tone to the chapters devoted to the sexual disorders of the female and special attention has been given to the injuries to the female genitals during coitus,—a subject which has been sadly overlooked by most other writers who, if they mention them at all, do so in a most superficial manner.

The author's presentation of the anatomy and physiology of the sexual apparatus has been much improved and is given in more extended form than in the previous editions. Many new illustrations have been added, and several new sections have been introduced which properly belong to a book of this character. Among them are tests for semen; composition of the normal prostatic fluid; clitoris crisis and the use of the x-ray in this special class of work. Stricture, varicocele, and posterior urethritis are given the careful consideration which is rightfully expected from a man of Taylor's knowledge of these subjects. The entire treatise is marked by a conservatism of tone, a prevalence of common sense, and an absence of fad worship and fancy adoration which are genuinely refreshing. The treatments outlined are based on common sense. The illustrations are excellent and helpful.—N. W. W.

